# Computer-aided studies of molecular structure-comparison of measured and computed ECD spectra

**DOI:** 10.1186/1758-2946-4-S1-P27

**Published:** 2012-05-01

**Authors:** Richard Moha, Verena Gossen

**Affiliations:** 1 Institute of Organic Chemistry, RWTH Aachen University, Landoltweg 1, 52074 Aachen, Germany

## 

**Figure 1:**
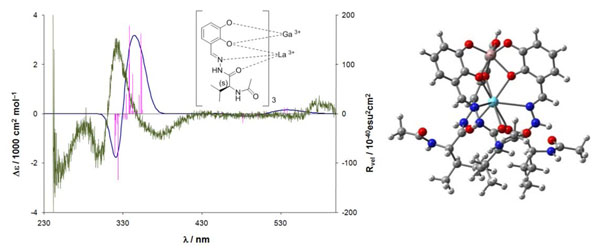
Left: Comparison of the measured CD spectrum in DMSO and the calculated spectrum of the complex, right: geometrically optimized structure (CAM-B3LYP).

ECD spectroscopy is an important instrument not only for the analysis of chirality but also for the study of conformational aspects of organic compounds [1]. In the present case, we studied helical complexes with different metals in the Λ or Δ form. ECD spectra of the complexes measured in DMSO (Figure 1) are compared to their calculated counterparts revealing conformational aspects of the structure. The structures of the complexes were optimized with the CAM-B3LYP functional [2], the SDD basis set and the effective core potential for the metal as implemented in the program package Gaussian09 [3]. TZVP was used for all other atoms. Subsequent time dependent DFT calculations were performed with the B3LYP functional.

Depending on the solvent and concentration, measured CD spectra are more or less noisy. Moreover, calculated spectra are frequently shifted to the blue while measured and calculated Δε might differ which complicates the analyses. Our newly coded *Spectra Curve Manager *[4] partly solves these problems. First the program subtracts the background from the measured raw spectrum. Subsequently, a graphical interface analyses differences between the measured and calculated spectra. Smoothing of the experimental spectra is performed by numerical algorithms, and the shift of the spectrum is minimized by displacement of the calculated spectra along the λ-axis. The Δε can be fitted as well. These combined methods facilitate comparison of measured and calculated spectra and, therefore, analysis of experimental results.
